# Mechanisms of Energy Metabolism in Skeletal Muscle Mitochondria Following Radiation Exposure

**DOI:** 10.3390/cells8090950

**Published:** 2019-08-21

**Authors:** Eun Ju Kim, Minyoung Lee, Da Yeon Kim, Kwang Il Kim, Jae Youn Yi

**Affiliations:** 1 Division of Basic Radiation Bioscience, Korea Institute of Radiological & Medical Sciences, Seoul 01812, Korea; 2 Radiological & Medico-Oncological Sciences, University of Science & Technology, Daejeon 34113, Korea; 3 Division of Radiation Research Infrastructure Operation, Korea Institute of Radiological & Medical Sciences, Seoul 01812, Korea

**Keywords:** ionizing radiation, mitochondrial bioenergetics, AMPK, PGC1, CPT1

## Abstract

An understanding of cellular processes that determine the response to ionizing radiation exposure is essential for improving radiotherapy and assessing risks to human health after accidental radiation exposure. Radiation exposure leads to many biological effects, but the mechanisms underlying the metabolic effects of radiation are not well known. Here, we investigated the effects of radiation exposure on the metabolic rate and mitochondrial bioenergetics in skeletal muscle. We show that ionizing radiation increased mitochondrial protein and mass and enhanced proton leak and mitochondrial maximal respiratory capacity, causing an increase in the fraction of mitochondrial respiration devoted to uncoupling reactions. Thus, mice and cells treated with radiation became energetically efficient and displayed increased fatty acid and amino acid oxidation metabolism through the citric acid cycle. Finally, we demonstrate that radiation-induced alterations in mitochondrial energy metabolism involved adenosine monophosphate-activated kinase signaling in skeletal muscle. Together, these results demonstrate that alterations in mitochondrial mass and function are important adaptive responses of skeletal muscle to radiation.

## 1. Introduction

Ionizing radiation (IR) is used as a treatment for many cancers and is an important diagnostic tool, but it is also a genetic toxicant that can negatively affect various cellular processes [1]. Fortunately, improvements in the ability to detect and treat cancer have resulted in a 60% survival rate of five years after cancer diagnosis, and about two-thirds of these survivors are receiving radiation therapy. Therefore, the late effect of radiation is an area of clinical interest [2]. IR also induces factors that are important in modulating mitochondrial biogenesis, which is related to cell survival: mitochondria have also been reported to be the primary target for radiation-induced apoptosis [3]. In addition, this organelle may play a role in radiation-induced intra- and intercellular signaling [4,5]. A subcellular proteomic analysis further revealed that proteins involved in energy metabolism processes are regulated by IR exposure in vivo [6]. However, the effects of IR on mitochondria have been much less intensively investigated than those on the cell nucleus.

Proper control of mitochondrial DNA (mtDNA) copy numbers is believed to be important for normal cell function [7]. An increase in the mtDNA copy number after radiation stimulation, termed “mitochondrial polyploidization” [8], is believed to be a compensatory mechanism or an adaptive response of mitochondria to maintain function in post-irradiated cells and malignantly transformed progeny that survive after radiation exposure [6,9]. The benefit of such an increase in the mtDNA copy number after irradiation is currently a matter of debate. In addition, mitochondria play an important role in the regulation of several cellular functions, including stress response, apoptosis, and metabolic processes such as gluconeogenesis and β-oxidation, and are sensitive to radiation and may have radiation damage within hours after exposure [10].

The increase in mtDNA content after irradiation may thus lead to the overproduction of mitochondrially encoded subunits. Notably, peroxisome proliferator-activated receptor coactivator 1 (PGC-1) is also associated with mitochondrial proliferation/numbers [11]. PGC-1, a transcriptional coactivator that is essential for mitochondrial biosynthesis, activates genes that regulate energy homeostasis and metabolism. In this context, PGC-1 modifies the metabolic rate and expression of genes involved in gluconeogenesis, fat oxidation, and mitochondrial biosynthesis [12]. PGC-1 also plays a key role in the oxidative metabolism of brown fat and muscle by increasing mitochondrial biogenesis and augmenting the expression of uncoupling proteins (UCPs) and enzymes of the electron transport system [13].

Adenosine monophosphate-activated kinase (AMPK) was first identified as a kinase that functions to inhibit fatty acid synthesis through phosphorylation and inactivation of acetyl coenzyme A carboxylase (ACC). Phosphorylation of ACC activates carnitine palmitoyl transferase 1 (CPT-1), thereby promoting the shuttling of fatty acids into the mitochondrial matrix for β-oxidation. AMPK is also known to enhance oxidative phosphorylation (OXPHOS) by promoting expression of the mitochondrial enzymes malate and succinate dehydrogenase [14]. AMPK, one of the most potent regulators of PGC-1 activity, can act through the phosphorylation of PGC-1 to increase both the charge and transcription of PGC-1. AMPK requires PGC-1 for many of its most important effects on mitochondrial gene expression in skeletal muscle, both in culture and in vivo [13,15].

Cell survival and growth require metabolic pathways that produce energy, precursors for macromolecular synthesis, and substrates for other essential functions [16]. The major nutrient substrates glucose, glutamine, and fatty acids can be completely oxidized to CO_2_ and H_2_O via the tricarboxylic acid (TCA) cycle. This requires operation of the mitochondrial electron transport chain (ETC), which couples a reaction using oxygen as a terminal electron acceptor to the production of adenosine triphosphate (ATP) through OXPHOS. Since living cells do not store ATP, they must produce it continuously and on demand, and therefore they constantly consume oxygen and fuel substrates [17]. Accordingly, understanding the molecular and bioenergetics events that promote early and late oxidative stress in irradiated cells/tissues will be informative for counteracting the adverse health effects of IR [18].

In mammals, muscles make up almost half of body weight, but it is common that they are generally resistant to radiation and are not an important part of body changes following irradiation [19]. However, transient weakness (a type of injury) and metabolic disorders have been observed in animals after irradiation [20], and childhood radiation therapy can cause muscle atrophy, weakness, impaired mobility, and fibrosis [2,21]. There is currently no information about the effects of IR on mitochondrial energy metabolism in skeletal muscle [22]. Thus, we hypothesized that IR-induced changes in mitochondrial energy metabolism include the activation of AMPK, PGC-1, CPT-1, and UCP-2 and play a role in maintaining mitochondrial activity and biogenesis in skeletal muscle. To this end, we examined IR regulation of potential roles in maintaining mitochondrial energy metabolism and mitochondrial homeostasis. Using animal and cell culture models, we observed that mitochondrial substrate utilization and intracellular metabolic activity were reprogrammed upon radiation exposure. In particular, we found that the production of acetyl-coenzyme A (CoA) from pyruvate was decreased by IR, which was a compensatory effect of using glutamine and fatty acids to supply α-ketoglutarate and palmitate in the TCA cycle. Taken together, these data highlight the capacity of IR to modulate mitochondrial activity in skeletal muscle.

## 2. Materials and Methods

### 2.1. Animals

Imprinting control region (ICR)-strain male mice (6–8 weeks old; average weight 22 ± 2 g) purchased from Koatech Co. (Pyeongtaek, South Korea) were maintained under specific pathogen-free conditions in a 12:12-h light–dark cycle (lights on from 8:00 a.m. to 8:00 p.m.) at a controlled temperature (22 ± 3 °C) and humidity (50% ± 20%). Mice were fed standard animal food pellets and tap water ad libitum and were acclimatized to laboratory conditions for 1 wk before use. All animal experiments were conducted according to our institution’s guidelines for the ethical use of animals, with the approval of the Institutional Animal Care and Use Committee of the Korea Institute of Radiological and Medical Sciences (KIRAMS; approved protocol KIRAMS 2014–17). Animals were euthanized in a CO_2_ chamber at a low flow rate (20–30% of the volume of the cage per minute), and blood and muscle tissues were collected. Muscle tissue samples, collected according to their muscle type, were fixed overnight in 10% buffered formalin, embedded in paraffin, sectioned for immunohistochemistry (IHC), snap-frozen in liquid nitrogen, and stored at -80 °C. Samples were subsequently analyzed for mRNA and protein expression.

### 2.2. Cell Culture

Mouse C2C12 skeletal myoblast-derived cells (ATCC; Manassas, VA, USA) were cultured in Dulbecco’s Modified Eagle Medium (DMEM) supplemented with 10% fetal bovine serum (FBS) and 1% antibiotic mixture in a humidified atmosphere of 95% air and 5% CO_2_ at 37 °C. Differentiation of C2C12 myoblasts was induced by transferring confluent cells to low-serum (1% FBS) DMEM and culturing for 4 to 5 days to allow the formation of myotubes. The differentiation medium was changed every 48 h.

### 2.3. Irradiation

Total body irradiation (TBI) of animals was accomplished at room temperature using an X-RAD 320 X-ray source (Precision X-Ray; North Branford, CT, USA) operated at 260 kVp and 10 mA using an F1 filter (2 mm aluminum) at a dose rate of 2 Gy/min. The reference dose rate was set to realistic radiation conditions in air on the animal plate with a UNIDOSE^®^ universal dosimeter (PTW; Freiburg, Germany). Each mouse was kept in a perforated plastic container. Mice undergoing radiation exposure were placed on a rotating platform to ensure even dose delivery to all tissues. Cells were irradiated at a dose rate of 3.81 Gy/min using a ^137^Cs gamma radiation source (Atomic Energy of Canada; Mississauga, ON, Canada) and then assessed at time points indicated in the text.

### 2.4. ELISA

Mouse blood samples were obtained and serum was immediately separated by centrifugation at 3000 rpm for 15 min at 4 °C. Muscle tissue samples (snap-frozen in liquid nitrogen) and C2C12 myotubes were disrupted using a TissueLyser II (Qiagen; Hilden, Germany) and homogenized in the extraction solution provided in the assay kit. Samples were deproteinized using 10-kDa molecular weight cut-off spin columns (Biovision; Milpitas, CA, USA). Assay kits were used to measure concentrations of lactate, pyruvate, acetyl-CoA, malate, glutamine, α-ketoglutarate (Biovision), and NAD^+^/NADH (Bioassay System; Hayward, CA, USA) according to the manufacturer’s instructions.

### 2.5. Reverse Transcription Polymerase Chain Reaction (PCR) and Real-Time PCR

Total RNA was isolated using the QIAzol reagent (Qiagen) and quantified by formaldehyde–agarose gel electrophoresis. Single-strand cDNA was synthesized from total RNA (2 μg) using 0.27 μg of oligo dT and amfiRivert reverse transcriptase (GenDEPOT; Katy, TX, USA). The desired cDNA fragments were amplified by real-time quantitative PCR (qPCR) using the primers listed in Table 1. Reactions were carried out in 20-μL reaction volumes containing 1 μL cDNA, 5× Hot FIREPol evaGreen qPCR Supermix (Solis BioDyne; Tartu, Estonia), and the relevant primers.

### 2.6. Measurement of Relative mtDNA Content

Total DNA was extracted from mouse muscle tissue and C2C12 myotubes using a QIAamp DNA Mini Kit (Qiagen), and mtDNA was measured by assessing the relative levels of ND2 (Table 1) and genomic DNA (gDNA) in extracts of total DNA using PCR analyses. Reactions were carried out in 20-μL reaction volumes containing 10 ng DNA, 5× Hot FIREPol Blend Master Mix (Solis BioDyne), and the relevant primers. Amplified products were resolved on ethidium bromide (0.1 μg/mL)-stained 2% agarose gels, visualized using UV spectrophotometry, and quantified with ImageJ software.

### 2.7. Immunohistochemistry

Paraffin-embedded sections were deparaffinized with xylene and dehydrated with an increasing ethanol series. Endogenous peroxidases were quenched by brief exposure to 3% H_2_O_2_, and antigen retrieval in citrate buffer was used to enhance the signal. For immunohistochemistry, specimens were incubated overnight at 4 °C with primary antibodies against COXIV (1:100 dilution; Abcam; Cambridge, United Kingdom) and then with a fluorescein isothiocyanate (FITC)-conjugated secondary antibody (1:200 dilution; BD Transduction Laboratories; Lexington, KY, USA) for 1 h at room temperature in the dark. Specimens were treated with 2 μg/mL propidium iodide (PI) in phosphate-buffered saline (PBS) for 5 min, mounted on glass slides, and observed under a Zeiss LSM 510 META confocal microscope (Carl Zeiss, Oberkochen, Germany).

### 2.8. Immunocytochemistry

C2C12 myoblasts were seeded on cell culture slides (SPL Life Sciences; Pochen-si, Gyeonggi-do, Korea) and allowed to form myotubes under the differentiation-inducing conditions described above. After treatment, myotubes were rinsed with PBS and immersed in 70% ethanol overnight. Cell culture slides were rinsed with PBS and fixed in 4% paraformaldehyde for 15 min at room temperature. This was followed by permeabilization with 0.1% Triton X-100 for 10 min and rinsing with PBS. Cells were blocked through incubation with sterile PBS containing 2% bovine serum albumin (BSA) for 1 h. Thereafter, cells were rinsed with PBS/0.1% BSA and incubated overnight at 4 °C with primary antibodies against COXIV (1:100 dilution; Abcam) and then with a FITC-conjugated secondary antibody (1:200 dilution; Invitrogen; Carlsbad, CA, USA) for 1 h at room temperature in the dark. Specimens were treated with 2 μg/mL PI in PBS for 5 min, mounted on glass slides, and observed under a Zeiss LSM 510 META confocal microscope (Carl Zeiss).

### 2.9. Western Blot Analysis

Total cell lysates were prepared in lysis buffer (50 mM Tris pH 7.4, 150 mM NaCl, 5 mM EDTA, 1% Triton X-100, 1% sodium deoxycholic acid, 30 mM Na_2_HPO_4_, 50 mM NaF, 1 mM Na_3_VO_4_) containing freshly added protease inhibitor cocktail (GenDEPOT). Nuclear, mitochondrial, and plasma membrane extracts were prepared using an NE-PER Nuclear and Cytoplasmic Extraction Kit (Pierce), a Mitochondria Isolation Kit (Abcam), and a Plasma Membrane Protein Extraction Kit (Abcam), respectively. Extracts (30 mg) were mixed with sodium dodecyl sulfate (SDS) sample buffer, boiled for 5 min, separated by SDS-PAGE (polyacrylamide gel electrophoresis) on 8% or 10% gels, and transferred to nitrocellulose membranes (MilliporeSigma, Burlington, MA, USA). Blots were incubated with primary antibodies against COXIV (ab14744), CPT-1 (ab128568) (Abcam), phospho-ACC (Ser79) (#3661), ACC (#3662), phospho-AMPK (Thr172) (#2531), AMPK (#2532), EGFR (#2232) (Cell Signaling Technology; Danvers, MA, USA), Glut-1 (sc-7903), Glut-4 (sc-7938), UCP-2 (sc-6525), Bcl-2 (sc-7382), U1 SnRNP 70 (sc-390899), and/or α-actin (sc-32251) (Santa Cruz Biotechnology; Santa Cruz, CA, USA), as described in the text.

### 2.10. Fluorometric and Fluorescence Microscopic Measurement of ROS Generation

C2C12 myoblasts were seeded on cell culture slides (SPL Life Sciences) and were allowed to form myotubes under differentiation-induced conditions, as described above. After treatment, the medium was removed, and prewarmed medium containing 200 nM MitoTracker Green (Invitrogen) was added. Myotubes were then incubated for 45 min in a humidified 5% CO_2_ atmosphere at 37 °C and fixed in 3.7% formaldehyde in prewarmed media. Myotubes were coverslip-mounted, cured, and imaged using a fluorescence microscope (Olympus, Tokyo, Japan). Medium containing MitoTracker Green was removed, and cells were suspended in prewarmed media. Group mean fluorescence was measured using a SpectraMax M2 Microplate reader (Molecular Devices; San Jose, CA, USA). For measurement of ROS generation, cells were incubated with 10 μM 2′,7′-dichlorodihydrofluorescein diacetate (DCF-DA; Sigma-Aldrich; St. Louis, MO, USA) for 30 min, and the intensity of DCF-DA fluorescence was determined using a fluorescence microplate reader.

### 2.11. Seahorse XF24 Metabolic Flux Analysis

Post-IR, the culture medium was removed and replaced with Seahorse XF Medium containing 25 mM glucose and 4 mM glutamine (CO_2_-free) and was incubated at 37 °C. Cellular oxygen consumption rate (OCR) and extracellular acidification rate (ECAR) were measured as described by the manufacturer. Optimization of reagents was performed using the Seahorse XF Mito Stress Test kit (Agilent; Santa Clara, CA, USA), applying the protocol and algorithm in the XF24 analyzer. Glycolysis was measured using a Seahorse XF Glycolysis Stress Test kit (Agilent), as described by the manufacturer, and is presented as ECAR. Three major fuel sources (glucose, glutamine, and fatty acids) were measured using a Seahorse XF Mito Fuel Flex Test kit (Agilent), as described by the manufacturer, and are presented as OCR. The Seahorse XF24 Extracellular Analyzer was run using 8-min cyclic protocol commands (mix for 3 min, let stand 2 min, and measure for 3 min) in triplicate.

### 2.12. Measurement of [^18^F]- Fluorodeoxyglucose (FDG) Uptake

On the day of the experiment, 2 mL of uptake medium (DMEM with 1 mg/mL glucose) containing 74 kBq (2 μCi) of [^18^F]-FDG was added to each well after rinsing the wells once in cold uptake medium. Myoblasts were incubated at 37 °C for 1 h to allow FDG to accumulate in cells. After washing twice in cold PBS, cells were lysed with 0.2% SDS, and radioactivity was immediately measured using a 1480 WIZARD gamma counter (PerkinElmer; Waltham, MA, USA).

### 2.13. Measurement of ATP

ATP was measured using an ATP Determination kit (Invitrogen) as described by the manufacturer. Briefly, myotubes were homogenized in lysis buffer (50 mM Tris-HCl pH 7.5, 1% Triton X-100, 0.1% SDS, 150 mM NaCl) freshly supplemented with the protease cocktail inhibitor Complete (GenDEPOT). ATP levels were assessed using a Micro-Lumat Plus LB96V fluorescence plate reader (Berthold Technologies; Bad Wildbad, Germany) and normalized to total protein, determined using a Bradford-based assay (GenDEPOT).

### 2.14. Statistical Analysis

Data are expressed as means ± standard deviations (SDs) of at least three independent experiments. Comparisons between two groups were analyzed by unpaired two-tailed Student’s *t*-tests, performed using Excel (Microsoft). A *P*-value <0.05 was regarded as significant (individual *P*-values are indicated in figure legends).

## 3. Results

### 3.1. Effects of Irradiation on Mouse Skeletal Muscle Tissue and Body Fluid Energy Homeostasis

**Figure 1:**
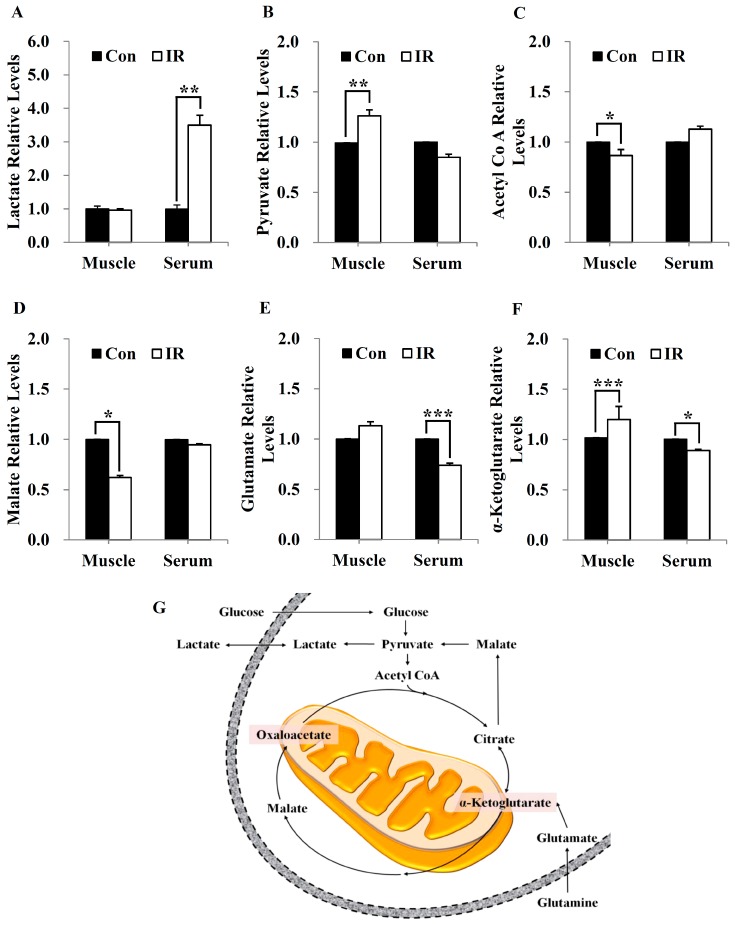
Radiation exposure altered mitochondrial metabolic substrate utilization in skeletal muscle. (**A**–**F**) Skeletal muscle and serum lactate (**A**), pyruvate (**B**), acetyl-CoA (**C**), malate (**D**), glutamate/glutamine (**E**), and α-ketoglutarate (**F**) were quantified in a control and (24 h after) in 2-Gy ionizing radiation (IR)-treated imprinting control region (ICR) mice using a metabolic assay kit and normalized to cellular protein content. Data are presented as means ± SD (*n* = 4; * *P* < 0.05, ** *P* < 0.01, *** *P* < 0.001). (**G**) Schematic illustration of cellular metabolism pathways and assays of glucose, glutamine, and mitochondrial tricarboxylic acid (TCA) cycle bioenergetics.

To confirm the expected effects of IR on levels of TCA cycle intermediates in skeletal muscle tissues and body fluids of ICR mice, we performed a metabolic analysis of intracellular TCA cycle intermediates and glutamate/glutamine—two essential amino acids [23] that can be derived from TCA cycle intermediates—with or without irradiation. Twenty-four hours after 2 Gy of IR exposure, lactate levels increased in serum, but were not altered in skeletal muscle (Figure 1A), whereas acetyl-CoA and malate levels decreased in skeletal muscle, but not in serum (Figure 1C,D). In contrast, pyruvate levels and glutamate/glutamine and α-ketoglutarate production were increased by IR exposure in skeletal muscle, but were decreased in serum (Figure 1B,E,F).

### 3.2. Alterations in the Expression of Skeletal Muscle Genes Associated with Mitochondria Biogenesis and Mitochondrial Content in Mice Exposed to IR

**Figure 2:**
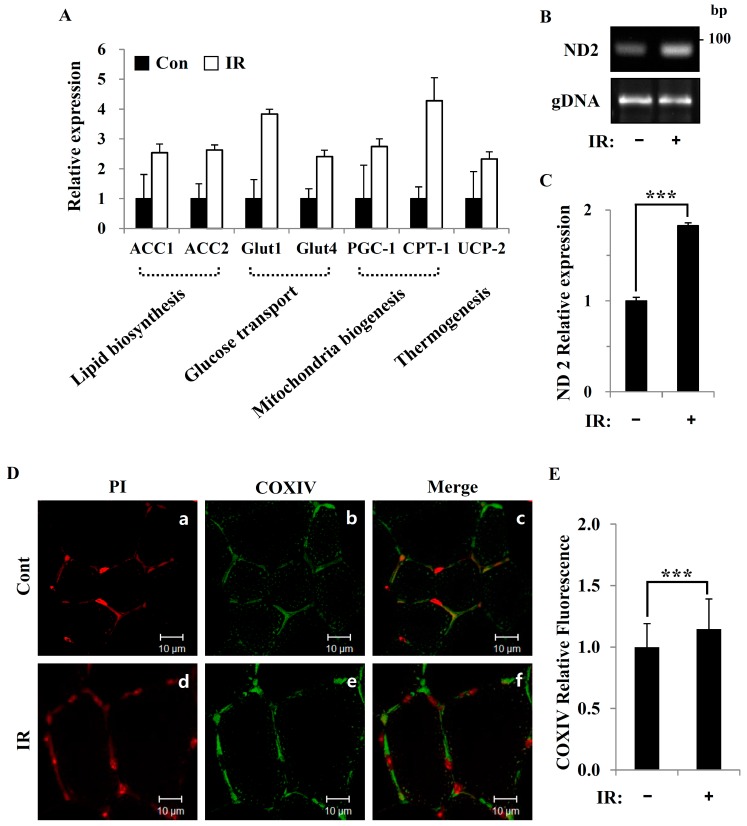
Radiation exposure altered the expression of mitochondrial biogenesis-related genes and mitochondrial mass in skeletal muscle: (**A**) qPCR analysis of ACC1, ACC2, Glut1, Glut4, PGC-1, CPT-1, and UCP-2 mRNA in skeletal muscle of control and (24 h after) 2-Gy IR-treated mice. Relative expression values were normalized to those in control mice. Data are presented as means ± SD (*n* = 4; *P* < 0.05). (**B**,**C**) Here, mtDNA content was analyzed by assessing the relative levels of ND2 and gDNA by conventional PCR (**B**) and qPCR (**C**) in the skeletal muscle of control and IR-treated mice. (**D**) Fixed skeletal muscle was stained with an antibody against COXIV (green): PI (red) was used to stain the nuclei. Scale bar: 10 μm. (**E**) COXIV (green) content, quantified using image J software and normalized to that in control skeletal muscle. Data are presented as means ± SD (*n* = 4; *** *P* < 0.001).

We next sought to more precisely understand the mechanisms through which radiation exposure increases mitochondrial biogenesis and mitochondrial content and to investigate their relationship to energy metabolism. We first found that mRNA levels of the lipid biosynthesis components ACC-1 and ACC-2 were increased by IR exposure in the gastrocnemius muscle of ICR mice. Additionally, transcript levels of genes associated with glucose transport (Glut-1 and Glut-4), mitochondrial biogenesis (PCG-1 and CPT-1), and thermogenesis (UCP-2) were increased in IR-exposed mice (Figure 2A). Moreover, mtDNA copy number, a commonly used marker of mitochondrial content, and the levels of ND2 (NADH hydrogenase 2) were similarly upregulated in mouse skeletal muscle following IR exposure (Figure 2B,C). We also measured the levels of the cytochrome c oxidase subunit, COXIV, a functional marker for maximal mitochondrial respiratory capacity. Indeed, IR-exposed mice showed a highly significant increase in COXIV expression compared to control mice (Figure 2D,E). Collectively, our findings demonstrate that radiation exposure increased mitochondrial biogenesis and improved mitochondrial function in mouse skeletal muscle.

### 3.3. Mitochondria Were Enriched and the Expression of Energy Metabolism-Related Genes Was Altered in IR-Treated C2C12 Myotubes

**Figure 3:**
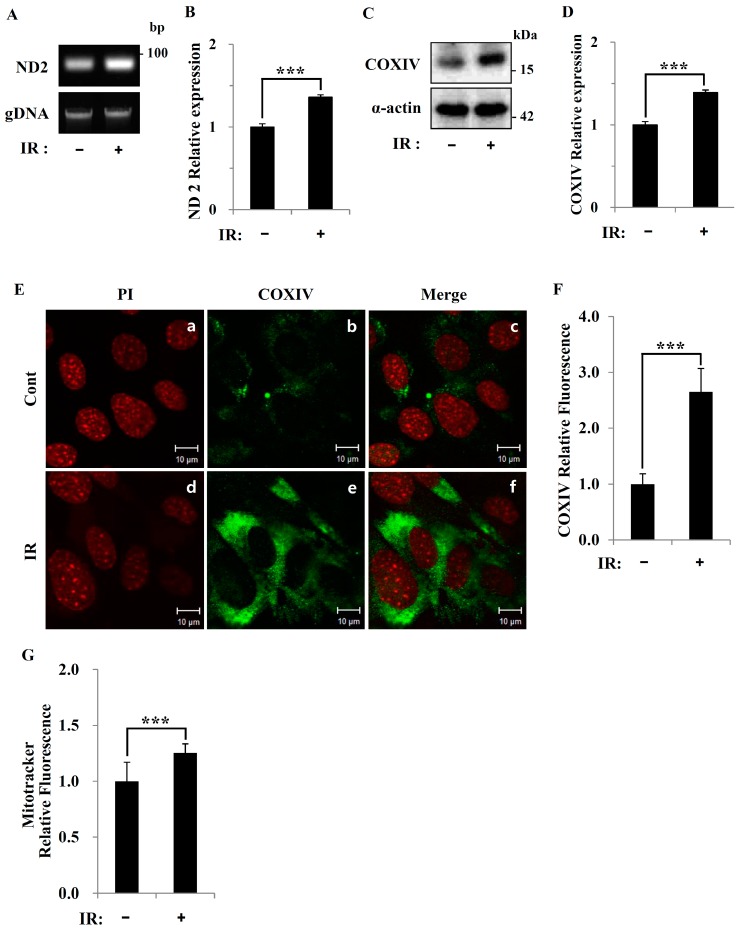

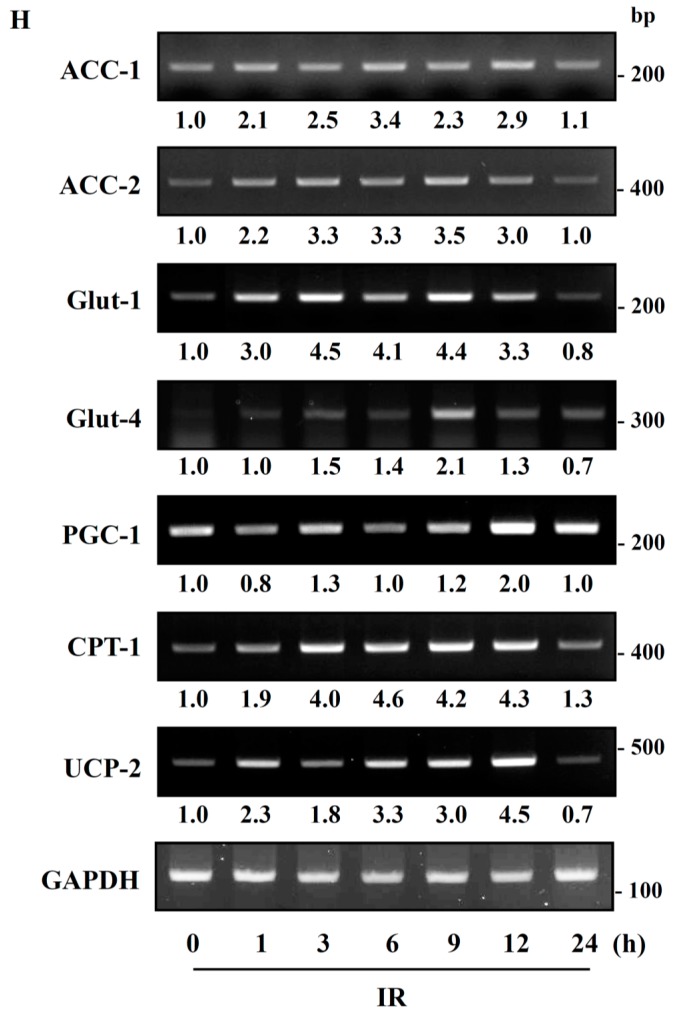
Increased mtDNA content and expression of mitochondrial biogenesis-related genes in response to radiation exposure: (**A**,**B**) mtDNA content in C2C12 myotubes of control and 6-Gy IR-treated mice after 24 h, analyzed by assessing the relative levels of ND2 and gDNA by conventional PCR (A) and qPCR (B). (**C**) Representative immunoblot for COXIV and α-actin in control and 6-Gy IR-treated C2C12 myotubes after 24 h. (**D**) COXIV content quantified using image **J** software. (**E**) Fixed C2C12 myotubes were stained with an antibody against COXIV (green): PI (red) was used to stain the nuclei. Scale bar: 10 μm. (**F**) COXIV (green) content was quantified using image **J** software and normalized to that in control C2C12 myotubes. (**G**) Mitochondria content in control and 6-Gy IR-treated C2C12 myotubes after 24 h, quantified using the mitochondrial marker Mito Tracker Green. (**H**) Conventional PCR analysis of ACC-1, ACC-2, Glut-1, Glut-4, PGC-1, CPT-1, and UCP-2 mRNA in control and 6-Gy IR-treated C2C12 myotubes at the indicated times. Relative expression values were quantified using image **J** software and normalized to those in control C2C12 myotubes. Values are expressed as means ± SD (*n* = 3; *** *P* < 0.001).

To evaluate changes in mitochondria mass, we first cultured C2C12 cells, a murine skeletal myoblast cell line, with 1% fetal bovine serum for 5 d, conditions in which these cells fuse to form myotubes. Differentiation was confirmed by light microscopy, which showed that myoblasts elongated and fused to form multinucleated tubes (data not shown). Following IR treatment, C2C12 myotubes exhibited an increase in mtDNA content compared to the untreated controls (Figure 3A,B). Immunoblotting revealed that levels of mitochondrial COXIV protein were also increased in IR-treated C2C12 myotubes (Figure 3C,D), results that were confirmed by immunostaining (Figure 3E,F). Fluorometric and fluoromicroscopic detection of the mitochondrial marker, MitoTracker Green, showed that mitochondria content was significantly increased in IR-exposed C2C12 myotubes compared to control myotubes (Figure 3G). In time course experiments, ACC-1, ACC-2, Glut-1, CPT-1, and UCP-2 mRNA expression levels in C2C12 myotubes were altered 1 h after IR exposure and were steadily increased at 3-, 6-, and 12-h time points, whereas Glut-4 and PGC-1 mRNA expression levels were not significantly increased until 9 h after radiation exposure (Figure 3H).

### 3.4. Radiation Exposure Altered Mitochondrial Oxidative Metabolism in C2C12 Myotubes

**Figure 4:**
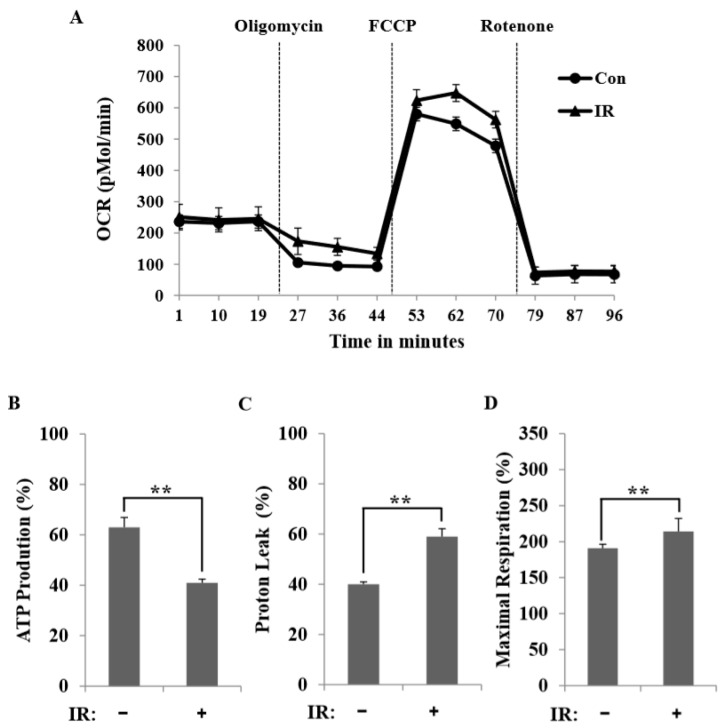
The generation of C2C12 myotubes revealed the ability of radiation exposure to improve mitochondrial respiration. (**A**) Kinetic oxygen consumption rate (OCR) responses of control and 6-Gy IR-treated C2C12 myotubes after 16 h to 2 μM oligomycin (ATP-coupled respiration), 5 μM FCCP (maximal respiratory capacity), and 1 μM rotenone (mitochondrial respiration). (**B**–**D**) Calculated ATP-coupled respiration (percent of oligomycin-sensitive OCR): (B) proton leak-linked respiration (percent of oligomycin-resistant, rotenone-sensitive mitochondrial OCR) (C) and maximal mitochondrial respiratory capacity (percent of rotenone-resistant OCR) (D) in control and 6-Gy IR-treated C2C12 myotubes. Each data point represents a mean ± SD (*n* = 3; ** *P* < 0.01).

To quantify changes in oxidative metabolism, we measured the OCR. We found that IR exposure altered the OCR in C2C12 myotubes (Figure 4A), as reflected in a higher mitochondrial respiratory capacity (determined by an FCCP-stimulated OCR) and an attenuated contribution of the OCR to basal ATP-coupled respiration (revealed by an oligomycin-sensitive OCR) (Figure 4B). Intriguingly, proton leak, measured as the difference between oligomycin-resistant and rotenone-sensitive OCRs, was markedly enhanced in IR-treated C2C12 myotubes, increasing to 63% of the baseline OCR compared to 42% in the control C2C12 myotubes (Figure 4C). Unexpectedly, IR exposure also significantly increased oxidative metabolism during maximal respiration compared to controls (Figure 4D). These results suggest that basal respiration in IR-treated C2C12 myotubes is largely uncoupled from the phosphorylation of ADP to ATP.

### 3.5. Radiation Exposure Altered Mitochondrial Glycolytic Metabolism in C2C12 Myotubes

**Figure 5:**
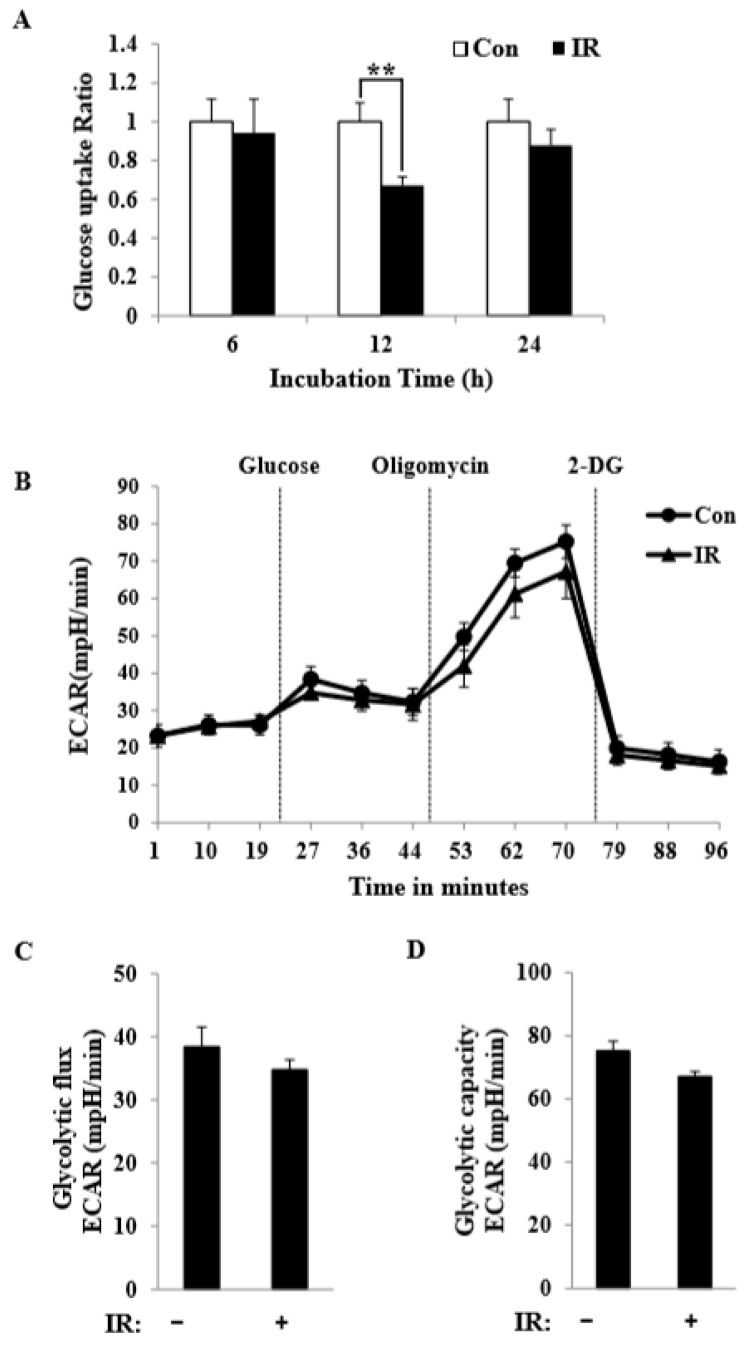
Measurement of glucose uptake, glycolysis, and glycolytic capacity of control and IR-treated C2C12 myotubes. (**A**) Glucose uptake was measured at the indicated times in control and 6-Gy IR-treated C2C12 myotubes and was normalized to protein concentration (means ± SD, *n* = 3; ** *P* < 0.01). (**B**) Kinetic extracellular acidification rate (ECAR) responses of control and 6-Gy IR-treated C2C12 myotubes after 16 h to 10 mM glucose, 2 μM oligomycin, and 50 mM 2-DG. (**C**,**D**) Calculated glycolysis (percent of glucose-sensitive ECAR) (C) and glycolytic capacity (percent of oligomycin-resistant, 2-deoxy-D-glucose (2-DG)-sensitive mitochondrial ECAR) (D) in control and 6-Gy IR-treated C2C12 myotubes. Each data point represents a mean ± SD (*n* = 3).

Cells that experience a loss of mitochondrial ATP production due to the inhibition of oxidative phosphorylation (e.g., due to low oxygen tension or inhibition by oligomycin) undergo a metabolic shift that results in augmented glycolytic flux—and thus greater production of ATP via glycolysis—that serves to maintain cellular ATP homeostasis [17]. Accordingly, we sought to determine whether the ability to maintain glucose homeostasis was lost in IR-exposed C2C12 myotubes. As expected, glucose levels in C2C12 myotubes were decreased under radiation exposure conditions (Figure 5A). We refer to this decreased glycolytic flux in response to a deficiency in mitochondrial ATP production as glycolytic capacity. To determine both glycolytic flux and glycolytic capacity of the same cell population in a single experiment, we measured ECAR while consecutively adding glucose, oligomycin, and 2-deoxy-D-glucose (2-DG) to the culture medium. As shown in Figure 5B, adding glucose to C2C12 myotubes triggered a glycolytic flux in C2C12 myotubes, as evidenced by the EACR value at 27 min. Subsequent addition of oligomycin caused a further increase in the ECAR value at 70 min, indicating an elevated glucose flux toward lactate and revealing the glycolytic capacity of C2C12 myotubes. The final addition of glycolysis inhibitor 2-DG abolished overall glycolysis. Glycolytic flux and glycolytic capacity calculated from the glycolysis experiment are shown in Figure 5C,D. Glycolysis and glycolytic capacities in IR-treated C2C12 myotubes (35 ± 3.1 and 67 ± 1.4 mpH/min, respectively) were much lower than those in control C2C12 myotubes (38 ± 5.1 and 75 ± 1.9 mpH/min, respectively).

### 3.6. Radiation Exposure Influenced Mitochondrial Substrate Utilization in C2C12 Myotubes

**Figure 6:**
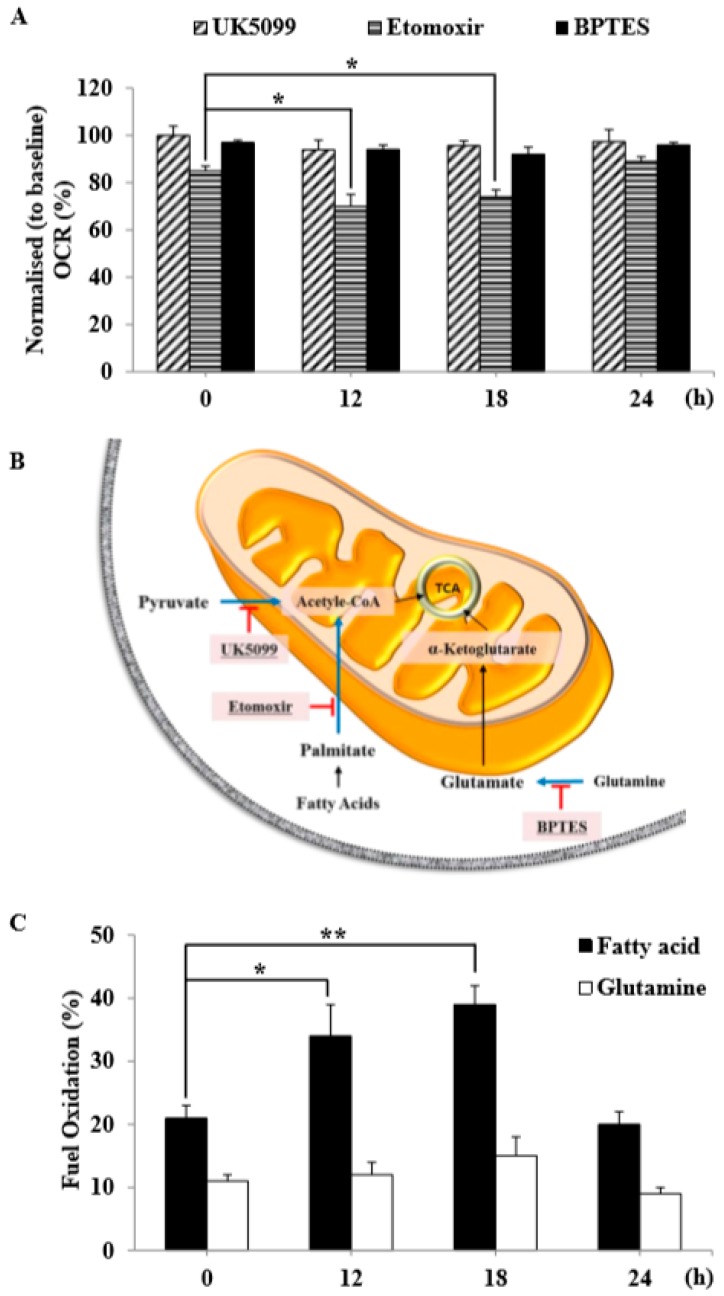
Pyruvate, fatty acid, and amino acids fueled mitochondrial metabolism following radiation exposure. (**A**) Kinetic maximal baseline-normalized OCR responses of control and 6-Gy IR-treated C2C12 myotubes after indicated times, with or without 15 μM UK5099 (blocks the mitochondrial pyruvate carrier, MPC), 30 μM etomoxir (inhibits carnitine palmitoyl-transferase 1A, CPT-1A), and 30 μM BPTES (allosteric inhibitor of glutaminase, GLS1). (**B**) Schematic illustration of cellular metabolism (glucose oxidation, long-chain fatty acid oxidation, and glutamine oxidation) pathways and assays for pyruvate, palmitate, and glutamate/glutamine. (**C**) Fuel oxidation was measured at the indicated times in control and 6-Gy IR-treated C2C12 myotubes. Each data point represents a mean ± SD (*n* = 3; * *P* < 0.05, ** *P* < 0.01).

To confirm the observed metabolic shifts in mitochondrial substrate utilization in an orthogonal manner, we quantified maximal respiration in control and IR-treated C2C12 myotubes in the absence or presence of UK5099 (covalently binds the mitochondrial pyruvate carrier (MPC) and blocks pyruvate transport), etomoxir (inhibits fatty acid oxidation at the CPT-1 step), and BPTES (inhibits glutamine oxidation via glutaminase). As shown in Figure 6A, maximal respiration was slightly decreased by UK5099 and BPTES treatment, with the most pronounced effect observed following the addition of etomoxir to IR-treated C2C12 myotubes at the 12- and 18-h time points. These results further highlight the increased reliance of C2C12 myotubes on fatty acid oxidation to fuel TCA metabolism upon radiation exposure (Figure 6C).

### 3.7. Mitochondrial Biogenesis in Response to Radiation Exposure Was Associated with the Activation of AMPK

**Figure 7:**
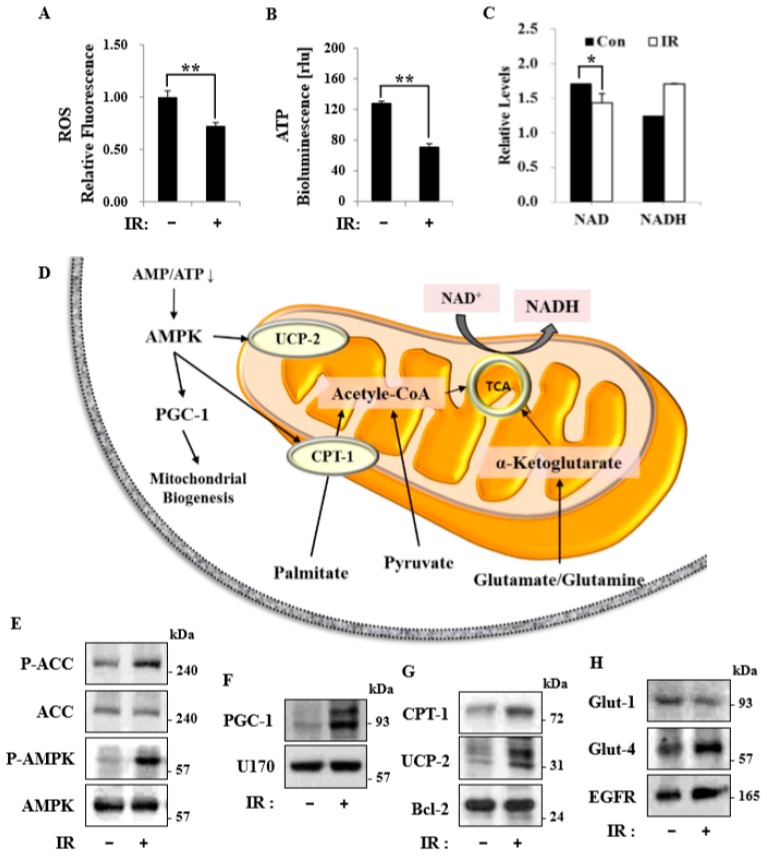
The activation of AMPK maintained bioenergetic homeostasis in C2C12 myotubes following radiation exposure. (**A**) Reactive oxygen species (ROS), (**B**) ATP, and (**C**) total NAD and NADH levels in C2C12 myotubes with or without 6-Gy radiation exposure were measured after 24 h and were normalized to protein concentration. Data are presented as means ± SD (*n* = 3; * *P* < 0.05, ** *P* < 0.01). (**D**) Schematic illustration of cellular molecular pathways. (**E**) Representative immunoblot for p-ACC (Ser79), total ACC, p-AMPK (Thr172), and total AMPK in control and 6-Gy IR-treated C2C12 myotubes after 24 h. (**F**) Representative immunoblot for nuclear PGC-1 and U170s in control and 6-Gy IR-treated C2C12 myotubes after 24 h. (**G**) Representative immunoblot for mitochondrial CPT-1, UCP-2, and Bcl2 in control and 6-Gy IR-treated C2C12 myotubes after 24 h. (**H**) Representative immunoblot for plasma membrane Glut1, Glut4, and EGFR in control and 6-Gy IR-treated C2C12 myotubes after 24 h.

Consistent with the observed metabolic shift, radiation exposure altered the production of reactive oxygen species (ROS), ATP pool size, and the NAD/NADH ratio at 18 h in C2C12 myotubes. Following radiation exposure, mitochondrial ROS (Figure 7A) and cellular ATP content (Figure 7B) were significantly decreased in C2C12 myotubes, and the NAD level declined (Figure 7C). AMPK is known to play an important role in cellular homeostasis, particularly under conditions of limited glucose availability [14]. AMPK and PGC-1 function as master integrators of cellular signals that regulate mitochondrial biogenesis, OXPHOS, adaptive thermogenesis, and fatty acid biosynthesis/degradation [24]. To investigate whether radiation-induced AMPK phosphorylation reflects the functional activation of AMPK (Figure 7D), we assessed phosphorylation of the AMPK substrate ACC in C2C12 myotubes. IR exposure induced the phosphorylation of ACC, an effect that required the expression of AMPK (Figure 7E). Subsequent analyses of protein expression in IR-treated C2C12 myotubes showed increased nuclear expression of the PGC-1 protein (Figure 7F), increased mitochondrial expression of the CPT-1 and UCP-2 proteins (Figure 7G), and increased plasma membrane expression of the Glut-4 protein (Figure 7H) compared to control C2C12 myotubes.

## 4. Discussion

The mitochondrion has traditionally been viewed as an organelle that functions as the “powerhouse” of mammalian cells, generating cellular energy in the form of ATP through the process of OXPHOS. It was first proposed by Harman [25] that defects in mitochondria are involved in severe clinical sequelae that affect the whole organism, or at least the central organs—the brain, heart, liver, and skeletal muscle—that have the highest energy consumption. Furthermore, a subcellular proteomic analysis has revealed that irradiation alters proteins involved in various energy metabolism-related processes. Therefore, more specific protection of mitochondria and mitochondrial energy metabolism would be expected to reduce damage to normal cells exposed to IR [6,26].

In this study, we tested radiation effects on mitochondrial energy metabolism in skeletal muscle, an organ with a high energetic demand that requires efficient mitochondrial function, and identified a number of important consequences of IR exposure. First, we provide strong evidence that mitochondrial metabolism is reprogrammed in response to radiation. Metabolic assays further indicated how metabolism was changed, showing that glutamine was metabolized to produce α-ketoglutarate, thereby enabling the TCA cycle to continue to produce energy. Surprisingly, radiation exposure also affected pyruvate, acetyl-CoA, malate, α-ketoglutamate, and glutamate/glutamine and significantly increased lactate secretion (Figure 1). We further observed a decrease in glucose uptake and glycolytic metabolism in skeletal muscle (Figure 5), suggesting that cells increase their reliance on glutamine anaplerosis when mitochondrial pyruvate transport is limited by reprogramming TCA metabolism. Second, based on previous work, we expected that radiation exposure would increase mitochondrial biogenesis, and we hypothesized that these effects would be dependent upon mitochondrial content. Further analyses revealed that radiation exposure increased mitochondrial mass in skeletal muscle (Figure 2 and Figure 3), suggesting that increased mitochondrial biogenesis may underlie the ability of IR to alter mitochondrial function. Finally, as might be expected, we observed an increase in AMPK activity following irradiation that correlated with increased mitochondrial metabolism, as assessed by both oxygen consumption and substrate utilization (Figure 4 and Figure 6). Taken together, our data suggest that IR exposure induced the phosphorylation of AMPK, which upregulated the mitochondria biogenesis-associated proteins PGC-1, UCP-2, and CPT-1 through the phosphorylation of ACC (Figure 7).

IR alters mitochondrial functions, increases mitochondrial oxidative stress, and causes specific changes in the expression of mitochondrial genes related to cell survival [3,27,28]. In sum, both genetic and metabolic susceptibility to IR greatly affect the outcome of radiation exposure. At the organelle level, mitochondria, as the major sites of oxidative metabolism, are almost certain to be affected. Although many studies have addressed the effects of IR on biochemical and morphological changes in mitochondria, studies of radiation-induced changes in the bioenergetics of this organelle are lacking. This is particularly important since mitochondrial structure and bioenergetics may be tightly linked [18]. In this study, we not only determined basal respiration, but also identified the type of exogenous nutrient substrates that are capable of being oxidized and the rates at which they can be oxidized under the experimental conditions used. Our results show that IR-exposed C2C12 myotubes possessed the ability to oxidize fatty acid and glutamine at higher rates than control C2C12 myotubes (Figure 6C). The synergistic effect of inhibiting these three mitochondrial substrate oxidation pathways highlights the plasticity of mitochondrial metabolism in respiring cells and independently demonstrates that radiation-exposed C2C12 myotubes employed fatty acid and amino acid oxidation to meet their bioenergetics demands. This suggests that C2C12 myotubes exposed to radiation shift away from glycolytic metabolism and adopt a more oxidative metabolism, perhaps through reprogramming of their metabolic network via certain epigenetic events.

Recent studies have shown that macroautophagy and chaperone-mediated autophagy are required to maintain the energy metabolism necessary to survive a variety of stimuli, including radiation [17]. Linking the observed changes in respiration and endogenous substrates, we speculate that autophagy may contribute, at least in part, to the provision of endogenous substrates in the form of fatty acids and amino acids. AMPK-dependent autophagy and/or mitophagy may represent important components of such metabolic control. Under energy stress conditions, mitochondrial homeostasis is ensured by AMPK-dependent mitophagy [29], which may serve to remove damaged, inefficient mitochondria following irradiation and thereby limit oxygen consumption [14]. Therefore, pharmacological activators of AMPK might act to protect against radiation damage. It further seems likely that unidentified substrates, substrate signaling networks, and pharmacological activators of AMPK in metabolic cells/tissues remain to be discovered and might serve as the basis for the development of strategies to defend against radiation-induced damage responses [30]. Mitochondrial dysfunction-associated senescence (MiDAS) [30], characterized by an imbalance in energy levels caused by a decreased NAD^+^/NADH ratio, is associated with cell cycle arrest resulting from stimulation of the cellular energy sensor, AMPK. Whether changes in AMPK and NAD^+^/NADH metabolism are causative factors or occur in parallel with IR-induced remodeling of the energy metabolism of skeletal muscle remains to be clarified [31].

## 5. Conclusions

The results of this study support the benefits of investigating the molecular mechanisms of mitochondria-related radiation responses as a prelude to developing therapeutic strategies for decreasing IR-induced damage. They also reinforce the need for further investigations into the effects of IR on mitochondria, including the mechanisms and biological significance of changes in mitochondrial mass and content in cells subsequent to IR exposure. Finally, it is likely that mitochondria are potential targets for radiation protection, a concept with broad relevance for clinical applications, the mitigation of environmental or industrial exposure, and space biology. Therefore, preclinical studies are needed to reveal the mechanisms that lead to the development of radiation-induced muscle disease and to help identify possible interventional goals.

## Figures and Tables

**Table 1:** Oligonucleotide primers.

Name	Sequence (5′→3′)	Product Size (bp)
ACC-1 (F)	GTC AGC GGA TGG GCG GAA TG	290
ACC-1 (R)	CGC CGG ATG CCA TGC TCA AC	
ACC-2 (F)	GCT GCG GTC AAG TGT ATG CG	460
ACC-2 (R)	CAC TGA TGC ATT TGC CCT GG	
GLUT-1 (F)	CGG GCC AAG AGT GTG CTA AA	290
GLUT-1 (R)	TGA CGA TAC CGG AGC CAA TG	
GLUT-4 (F)	CAC AGA AGG TGA TTG AAC AGA	310
GLUT-4 (R)	GTT AGC CCT GAG TAG GCG CC	
PGC-1 (F)	ACG AGG CCA GTC CTT CCT CC	270
PGC-1 (R)	AGC TCT GAG CAG GGA CGT CT	
CPT-1 (F)	CAG TCA GAG CAG CTA GGT GT	420
CPT-1 (R)	GCT CTC GAG GCT CAC TGA TT	
UCP-2 (F)	AAC AGT TCT ACA CCA AGG GC	470
UCP-2 (R)	AGC ATG GTA AGG GCA CAG TG	
GAPDH (F)	AAG GGC TCA TGA CCA CAG TC	160
GAPDH (R)	TTC AGC TCT GGG ATG ACC TT	
mtDNA ND2 (F)	CAC GAT CAA CTG AAG CAG CAA	76
mtDNA ND2 (R)	ACG ATG GCC AGG AGG ATA ATT	

## Abbreviations

IR, ionizing radiation; mtDNA, mitochondrial DNA; PGC-1, peroxisome proliferator-activated receptor coactivator 1; UCP-2, uncoupling protein 2; AMPK, adenosine monophosphate-activated kinase; ACC, acetyl coenzyme A carboxylase; CPT-1, carnitine palmitoyl transferase 1; EGFR, Epidermal growth factor receptor; OXPHOS, oxidative phosphorylation; TCA, tricarboxylic acid; ETC, electron transport chain; ATP, adenosine triphosphate; ND2, NADH hydrogenase 2; DCF-DA, 2′,7′-dichlorodihydrofluorescein diacetate; Glut, glucose transport; FCCP, Carbonyl cyanide-4-(trifluoromethoxy)phenylhydrazone; 2-DG, 2-deoxy-D-glucose; OCR, oxygen consumption rate; ECAR, extracellular acidification rates; MPC, mitochondrial pyruvate carrier; BPTES, Bis-2-(5-phenylacetamido-1,3,4-thiadiazol-2-yl)ethyl sulfide
